# Genomic selection improves survival time under high ammonia nitrogen stress in *Litopenaeus vannamei*

**DOI:** 10.1186/s12711-026-01081-6

**Published:** 2026-08-27

**Authors:** Shuo Fu, Yuan Zhang, Guangbo Wu, Chaoan Guo, Jianyong Liu

**Affiliations:** Fisheries College, Guangdong Ocean University, No. 1 Haida Road, Zhanjiang, 524088 Guangdong China

## Abstract

****Background**:**

*Litopenaeus vannamei* is an economically important species in global aquaculture. Enhancing its environmental resistance through selective breeding is important for improving the efficiency of intensive farming. Genomic selection (GS) can speed up genetic improvement, but it has not been widely used in commercial shrimp breeding because genotyping entire candidate populations is too expensive. In this study, we compared the predictive ability of six GS models with that of the traditional pedigree-based best linear unbiased prediction (PBLUP) model to evaluate the effect of GS on the high ammonia nitrogen resistance in *L. vannamei*. We then proposed and validated a cost-effective breeding strategy that combines BLUP method with GS in the progeny.

****Results**:**

The heritability of high ammonia nitrogen tolerance was 0.26 from the pedigree model, and 0.56–0.58 from genomic models. GS improved prediction accuracy by 32.45–39.25% compared with pedigree-based PBLUP. Progeny validation based on ssGBLUP showed that the high-resistance group had 11.38−11.82% longer survival time than the sensitive group, with a significant positive correlation between Genomic Estimated Breeding Values (GEBVs) and observed survival time.

****Conclusions**:**

The integration of BLUP with GS provides an economically feasible breeding strategy for shrimp. This approach balances accuracy and cost, making genomic information practical for commercial shrimp breeding.

**Supplementary Information:**

The online version contains supplementary material available at 10.1186/s12711-026-01081-6.

## Background

*Litopenaeus vannamei* is a key species in the global aquaculture industry, accounting for approximately 80% of global farmed shrimp production with an annual yield of more than four million tons [1]. This commercial success is supported by several key biological traits, such as fast growth, high resistance to intensive cultivation conditions, and strong osmoregulatory capabilities across a broad salinity spectrum.

Currently, intensive farming has become the main model for *L. vannamei* to improve the production efficiency. Nevertheless, this approach has a serious problem: the progressive accumulation of ammonia nitrogen in the farm water. Ammonia accumulation is a widespread and severe issue causing suppressed growth, impaired immunity, and massive economic losses in commercial aquaculture [2–4]. This increase in ammonia nitrogen concentration mainly comes from two sources: direct release of nitrogen-based wastes by the shrimp and the microbial mineralization of organic matter, such as residual feed and feces. Long-term exposure to high ammonia nitrogen levels causes severe physiological harm and is known to trigger oxidative stress-mediated pathology [5]. Moreover, ammonia poisoning disrupts basic body processes, including the speeding up of molting cycles and damage to their shells. [6–8]. These problems work together to weaken the organism’s immune system, leading to heightened susceptibility to disease and increased mortality during the farming period.

To reduce the severe economic losses caused by high ammonia nitrogen in *L. vannamei* aquaculture, the development of new, more resilient strains through selective breeding is a critical priority. While traditional breeding strategies, including mass selection and family-based programs [9], have yielded some improvements, they prove insufficient for complex environmental stress traits characterized by low heritability [10, 11]. For instance, the heritability for ammonia nitrogen resistance in *L. vannamei* has been estimated at low values of 0.13 and 0.17 [12], and similar limitations have been observed for dissolved oxygen tolerance, with a heritability of just 0.07±0.03 [13]. These findings match the inherent constraints of traditional methods, such as long breeding cycles and slower improvement over generations, which arise from polygenic regulation and genotype-by-environment interactions in stress-resistance traits. As a result, there is an urgent need to adopt advanced genomic tools to overcome these hindrances and accelerate genetic improvement [10, 11].

The advent of molecular marker-assisted selection (MAS) and genome-wide association studies (GWAS) has greatly improved the ability for precision breeding by allowing the direct selection of individuals carrying genetic markers connected to the desired traits [14]. In decapod crustaceans, these approaches have been widely applied to identify single nucleotide polymorphisms (SNPs) related to with key commercial traits, including growth, disease resistance, and environmental stress resistance [15–18].While these studies have reinforced the understanding that such traits are polygenic—controlled by numerous loci each with a small effect, they also expose a critical limitation of MAS and GWAS. By focusing on a restricted subset of statistically significant SNPs, these methods ignore the cumulative contribution of many minor effect loci, thereby leading to incomplete use of genomic information and consequently compromising the predictive accuracy for complex phenotypic traits [19, 20].

In contrast to MAS and GWAS, genomic selection (GS) is a more effective method for improving complex quantitative traits. By using genome-wide markers, GS calculates genomic estimated breeding value (GEBV) for each candidate, integrating the effects of all genotyped SNPs without requiring them to surpass a stringent significance threshold [19]. The efficacy of GS in accelerating genetic gain has been demonstrated across a diverse range of aquaculture species, including Zhikong scallop (*Chlamys farreri*) [21], Atlantic salmon (*Salmo salar*) [22], rainbow trout (*Oncorhynchus mykiss*) [23], Nile tilapia (*Oreochromis niloticus*) [24], Japanese flounder (*Paralichthys olivaceus*) [25], large yellow croaker(*Larimichthys crocea*) [26] and triangle sail mussel (*Hyriopsis cumingii*) [27], among others.

In *L. vannamei*, GS has been successfully implemented to improve growth performance [28, 29], resistance to White Spot Syndrome Virus (WSSV) [30]and Acute Hepatopancreatic Necrosis Disease (AHPND) [31], tolerance to high salinity [32], and feed efficiency traits [33]. To date, no study has examined the feasibility of GS for ammonia nitrogen resistance in shrimp. The objective of the present study was to assess the efficiency of GS for ammonia tolerance in *L. vannamei*. We compared predictive accuracy across multiple GS models versus conventional PBLUP via cross-validation and further validated selection effects through independent progeny challenge tests. To reduce genotyping costs for industrial promotion, we also explored a cost-effective breeding scheme integrating BLUP and GS, in which progeny GEBVs were calculated using a 50K SNP array and correlated with practical survival phenotypes. This combined strategy enables a feasible transition of shrimp breeding from conventional selection to GS.

## Methods

### Material collection

In March 2023, 51 families were established through incomplete diallel crossing design using breeders from the core breeding population maintained at the hatchery. This core population originated from five different geographical sources of shrimp and has been subjected to mild selection for growth traits over multiple generations. Larvae from each family were reared separately under standard conditions for two months, reaching an average body length of about 4 cm. From these families, 22 families that were established on the same day were selected for challenge testing. These 22 full-sib families were derived from 14 sires and 13 dams, and this mating structure included 12 half-sib families. Thirty individuals from each of the 22 families were tagged with visible implant elastomer (VIE) tags; individuals within the same family shared the same tag color, while those from different families had distinct colors. All shrimp were then reared together in a single tank to minimize environmental variation. After another two months, a total of 566 individuals (23–27 per family) were selected for the ammonia nitrogen stress test to assess resistance phenotypes.

### High ammonia nitrogen stress test

A population of 566 individuals was used for the stress experiment. Based on preliminary trials, the stress concentration was set at 85 mg/L ammonia nitrogen, which was our determined 96-h semi-lethal concentration ($LC_{50}$). For stress experiment, shrimp were placed in a 1.8 ×1.8 m test pool. After a five-day holding period, the concentration of ammonia nitrogen was adjusted to 85 mg/L by adding analytical-grade ammonium chloride. During the experiment, water was replaced daily, the water temperature was maintained at 25 ± 1.5 $^{\circ }$C, the pH was maintained at 8.1 ± 0.2, the salinity was maintained at 30 ± 1, and the dissolved oxygen was maintained at 6–8 mg/L. We checked for mortalities every hour and recorded the survival time for each shrimp. Shrimp were considered dead if they lay motionless at the bottom of the pool and did not respond to touch. Immediately after death, a muscle tissue sample was taken from each shrimp and stored in absolute ethanol for later DNA extraction. The experiment ended when all shrimp had died at approximately 160 h post-exposure.

### DNA extraction and genotyping

We ranked all shrimp by ammonia tolerance across the population and performed stratified random sampling across low, intermediate, and high phenotypic groups, selecting 200 individuals for DNA extraction. This ensured that the sampled population approximately followed a normal distribution of ammonia resistance. It should be noted that families 9 and 14 were excluded because their average body weights (13.5 g and 15.1 g, respectively) deviated markedly from the overall family mean of 9.26 g ± 1.50 g. Outliers were defined as values exceeding the overall family mean ± 2 standard deviations. Genomic DNA was extracted from muscle tissues using the EasyPure^®^ Genomic DNA Kit (TRANSGEN BIOTECH, China) following the manufacturer’s protocol. We checked DNA integrity on a 1% agarose gel. DNA concentration and purity were measured with a NanoDrop 2000 spectrophotometer (Thermo Fisher Scientific, Waltham, USA). Samples with absorbance ratios of A260/A280 = 1.8−2.0 and A260/A230 > 1.8 were considered high quality and stored at − 20 $^{\circ }$C.

Qualified samples were sent to Huazhi Biotechnology Co., Ltd. (Changsha, China) for resequencing, using the MGISEQ-2000 platform with a sequencing depth of 10× and a sequencing strategy of PE-150. Whole-genome resequencing was performed for the 200 shrimp individuals used in this study, alongside 400 additional conspecific individuals from a parallel project, forming a joint 600-individual resequencing dataset. Raw sequencing data were quality-controlled with FASTP. This process involved the removal of: (1) reads shorter than 100 bp; (2) reads containing adapter sequences; (3) reads with more than five “N” bases; (4) reads where over 50% of bases exhibited a quality score of 20 or below. SNP calling was performed using the Sentieon software by aligning the quality-controlled reads to the reference genome (NCBI assembly: GCF_042767895.1), resulting in 20,193,853 raw SNPs. A round of basic variant filtering was then performed on the 600-individual dataset using the parameters -max-missing 0.9 -maf 0.05 -max-alleles 2, yielding 19,205,386 filtered SNPs as the final delivered dataset. Subsequently, we extracted the genotype data of the 200 target individuals from the original variant call format (VCF) file, and conducted a second round quality control using PLINK v1.90 with the following criteria: (1) per-SNP missing rate *≤ * 10% (–geno 0.1); (2) minor allele frequency (MAF) *≥ * 0.01 (–maf 0.01); (3) exclusion of variants significantly deviating from Hardy-Weinberg equilibrium (HWE $\textit{p} < 1 \times 10^{-5}$, –hwe 1e-5). After this filtering step, a total of 3,276,958 variants were excluded, and 15,928,425 high-quality SNPs were retained for downstream analyses. Principal component analysis (PCA) was conducted on 200 shrimp individuals using Plink, and the resulting PCA outputs were visualized via the ggplot2 package in R (v4.3.1).

To ensure the consistency of pedigree data and genomic data, we adopted the SIBCOUNT and SIBREL methods to filter the samples [34], with all filtering processes conducted in the R environment. SIBCOUNT (Opposing Homozygote Count): For each pair of individuals recorded as full-sibs in the pedigree, we counted the number of loci with opposing homozygous genotypes. The observed counts were compared to theoretical expectations derived from allele frequencies. Pairs exceeding the threshold (mean + 5SD) were flagged as inconsistent. SIBREL (Relationship Comparison): We constructed a pedigree-based relationship matrix ($\mathbf{A}$) from the five-generation pedigree using the complete pedigree and a genomic relationship matrix ($\mathbf{G}$) using ASReml. For each full-sib pair, we computed the absolute difference between their genomic ($\mathbf{G}$) and pedigree ($\mathbf{A}$) relationships ($\Delta = |\mathbf{G}_{ij} - \mathbf{A}_{ij}|$). Pairs with *Δ > 0.5* were considered inconsistent. The union of both methods removed 2 individuals (1% of the genotyped population).

### Statistical methods

Heritability for high ammonia nitrogen resistance was estimated from variance components, using the following formula:

1 $$\begin{aligned} h^2=\frac{\sigma _u^2}{\sigma _u^2+\sigma _p^2+\sigma _e^2} \end{aligned}$$

where $h^2$ represents heritability, $\sigma _u^2$ represents the additive genetic variance of high ammonia nitrogen resistance trait, $\sigma _p^2$ denotes the common environmental (tank) variance, and $\sigma _e^2$ is the residual variance. For PBLUP, GBLUP, and ssGBLUP, variance components were obtained via a linear mixed model fitted with restricted maximum likelihood. For BayesA, BayesB, BayesC*π *, and Bayesian LASSO, variance components were estimated from posterior samples generated by Gibbs sampling in the BGLR package. At each iteration, marker effects were sampled and GEBVs were obtained as the sum of marker contributions across all loci. The additive genetic variance was calculated as the variance of GEBVs across individuals. Residual variance and common environmental variance were estimated simultaneously from their corresponding posterior distributions. Posterior means across retained iterations were used as final estimates of variance components, from which heritability was calculated. The $\mathbf{A}$ matrix was derived from pedigree information, the $\mathbf{G}$ matrix was constructed from 15,928,425 high-quality SNPs using the method described by Vanraden [35], and the $\mathbf{H}$ matrix was developed using both pedigree and genomic information (from the 15,928,425 SNPs) using the method described by Legarra et al. [36] The pedigree for $\mathbf{A}$ and $\mathbf{H}$ matrix construction covered five generations with a total of 332 individuals. Specifically, 200 individuals possessed both phenotypic and genotypic data, while the remaining 132 historical ancestors only had pedigree records and lacked phenotype and genotype data.

We compared the accuracy of seven different models for estimating breeding values: pedigree-based best linear unbiased prediction (PBLUP), genomic BLUP (GBLUP), single-step GBLUP (ssGBLUP), and four Bayesian models (BayesA, BayesB, BayesC*π * and Bayesian LASSO). The prediction model of PBLUP is as follows:

2 $$\begin{aligned} \mathbf{y} = \mathbf{X}\mathbf{b} + \mathbf{Z}\mathbf{u} + \mathbf{W}\mathbf{p} + \mathbf{e} \end{aligned}$$

Among them, $\mathbf{y}$ is the vector of phenotypes (survival time), $\mathbf{X}$ is the design matrix for fixed effects, including the covariate of body weight and body length, and $\mathbf{b}$ is the corresponding vector of fixed effect solutions. $\mathbf{u}$ is the vector of additive genetic effects (individual breeding values), following $\mathbf{u} \sim N(0, \mathbf{A}\sigma _{u}^{2})$, where $\mathbf{A}$ is the pedigree relationship matrix and $\sigma _{u}^{2}$ is the additive genetic variance; $\mathbf{Z}$ is the incidence matrix linking individuals to their additive genetic effects. $\mathbf{W}$ is the incidence matrix linking individuals to their respective tanks (common environmental groups), and $\mathbf{p}$ is the vector of random common environmental effects (tank effects), assumed $\mathbf{p} \sim N(0, \mathbf{I}\sigma _p^2)$, where $\sigma _p^2$ is the common environmental variance; $\mathbf{e}$ is the residual effect, following $\mathbf{e} \sim N(0, \mathbf{I}\sigma _{e}^{2})$, where $\mathbf{I}$ is the identity matrix. The prediction model of GBLUP is similar to that of PBLUP, but the pedigree-based $\mathbf{A}$ matrix is replaced by the genomic $\mathbf{G}$ matrix constructed from molecular markers. Similarly, the ssGBLUP model uses the $\mathbf{H}$ matrix, which integrates both pedigree and genomic information.

All four Bayesian models use the following formula:

3 $$\begin{aligned} \mathbf{y} = \mathbf{X}\mathbf{b} + \mathbf{Z}\boldsymbol{\alpha } + \mathbf{W}\mathbf{p} + \mathbf{e} \end{aligned}$$

where $\mathbf{y}$ is the phenotype vector; $\mathbf{b}$ is the vector of fixed effects, including the population mean and the body weight and length covariates, and $\mathbf{X}$ is the corresponding design matrix; $\mathbf{Z}$ is the matrix of centered and scaled genotypes, and $\boldsymbol{\alpha }$ is the vector of SNP effects, such that $\mathbf{Z}\boldsymbol{\alpha }$ represents the vector of additive breeding values; $\mathbf{p}$ is the vector of random common environmental effects (tank effects), assumed to follow a multivariate normal distribution:

$$ \mathbf{p} \sim N(0, \mathbf{C}\sigma _p^2) $$

where $\mathbf{C}$ is a block-diagonal covariance matrix defined as:

$$ C_{ij} = {\left\{ \begin{array}{ll} 1 & \text {if individuals } i \text { and } j \text { are from the same tank} \\ 0 & \text {otherwise} \end{array}\right.} $$,

and $\mathbf{W}$ is the incidence matrix linking records to tank effects; $\mathbf{e}$ is the residual vector following a normal distribution $\mathbf{e} \sim N(0, \mathbf{I}\sigma _e^2)$, where $\mathbf{I}$ is the identity matrix. The differences among different Bayesian models lie in their different assumptions about the distribution of marker effects ($\alpha _j$). BayesA assumes that all markers have an effect, and the variance for each effect follows an inverted chi-squared distribution. BayesB introduces an indicator parameter (*π *) for SNP effects, assuming a large proportion (*π *) of markers have no effect, while only a small proportion (1-*π *) have effects. The variances of these non-zero effects follow an inverted chi-squared distribution. BayesC*π * is similar to BayesB, but with the distinction that all non-zero effect SNPs are assumed to have a common variance, the value of which is updated by the algorithm. Similar to BayesA, Bayesian LASSO assumes all markers have effects but applies strong shrinkage to the marker effects using a Laplace prior [37, 38].

Analyses for PBLUP, GBLUP, and ssGBLUP were run using the ASReml-R package (v4.2.0.276) in R (v4.3.1). The Bayesian models were run using the BGLR package (v1.1.4) in R (v4.3.1). For the Bayesian analyses, we ran 20,000 Gibbs sampling iterations, discarding the first 5000 as burn-in.

### Cross-validation and comparison of correlation

In each validation, we used four subsets (80%) as the training set and the remaining one (20%) as the validation set, where the phenotype data was hidden. Given the limited number of families available in this study, partitioning was performed at the individual level rather than the family level, which allowed related individuals from the same family to appear in both the training and validation sets and may result in more optimistic accuracy estimates, particularly for genomic models. The five-fold cross-validation analysis was repeated 20 times, resulting in a total of 100 validation runs. Prediction accuracy was calculated as the Pearson correlation between the (G)EBVs of the validation set and the corresponding phenotypes adjusted for fixed effects ($y^*$), divided by the square root of the PBLUP heritability estimate ($\sqrt{h^2}$).

4 $$\begin{aligned} { \text {Accuracy} = \frac{r(\text {GEBV}, y^*)}{\sqrt{h^2}}} \end{aligned}$$

The prediction bias was assessed by the regression coefficient of observed phenotypes on predicted (G)EBVs. A regression coefficient of 1 indicates unbiased prediction, where the (G)EBVs accurately reflect the true breeding values. A coefficient greater than 1 suggests that the (G)EBVs are deflated (underestimated in magnitude), while a coefficient less than 1 indicates that the (G)EBVs are inflated (overestimated in magnitude). After estimating the breeding values for each individual using different predictive models, we calculated the Pearson correlations between the breeding values of these models and plotted a correlation heatmap using R software. To evaluate the consistency of model predictions under a realistic selection scenario, we also specifically analyzed the correlation of breeding values among the top 30% individuals with the longest survival times.

### Progeny validation

**Fig. 1:**
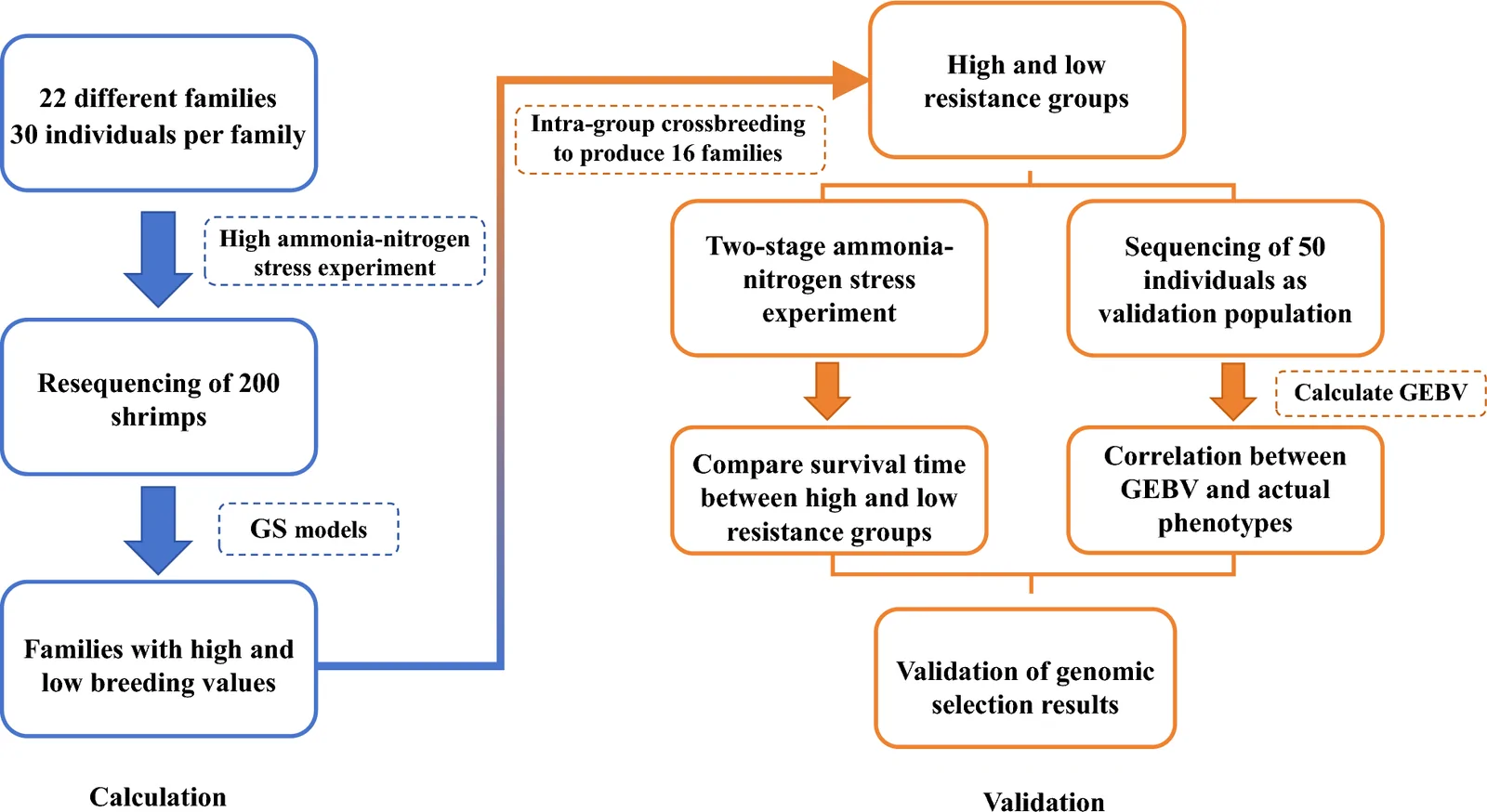
Schematic diagram of the experimental workflow

For further validation, based on the correlation between different GS models, we conducted a progeny test using ssGBLUP model, the general process is as shown in Fig. 1. Unlike conventional GS, instead of performing genotyping on candidate parents of progeny populations, we calculated the average GEBVs of different families in the reference population. The top 20% of families ranked by average GEBVs (4 families) constituted the high-resistance group, and the bottom 20% (4 families) constituted the sensitive group. Selfing and crossing were performed separately for each of the four selected families within each group to establish 16 families using complete diallel crosses. These 16 families from each group were mixed for standardized cultivation, giving rise to the offspring of the high-resistance group and the sensitive group.

After two months, 200 shrimp from each group were selected for the first-stage ammonia nitrogen stress test at 60 mg/L concentration, with mortality recorded every 6 h. For the second-stage stress, 120 shrimp from each group were tagged and co-reared in a common environment for three months to eliminate environmental effects, followed by a high ammonia nitrogen stress trial (160 mg/L) using the same methodology as “High ammonia nitrogen stress test” section, with observations every 2 h. Muscle tissues from the first 25 shrimp and the last 25 to die were preserved for DNA extraction. Genotyping was performed using a self-developed 50K liquid-phase breeding chip [39]. To ensure compatibility between the genotype data of the reference and validation populations, SNPs simultaneously present in both the whole-genome resequencing dataset and the 50K liquid-phase breeding chip dataset were retained. These markers were used to construct a new SNP dataset for GEBV estimation. The genotype data of validation population was integrated into the reference population for GEBVs calculation. The correlation between GEBVs and the actual survival time phenotypes was analyzed. As pedigree information for the validation population was unavailable, GBLUP model was employed for GEBVs estimation. We constructed Kaplan-Meier survival curves for both the high-resistance and sensitive groups across two independent validation stages and compared these curves using the Log-rank test.

## Results

### Descriptive statistics of phenotypic values

**Fig. 2:**
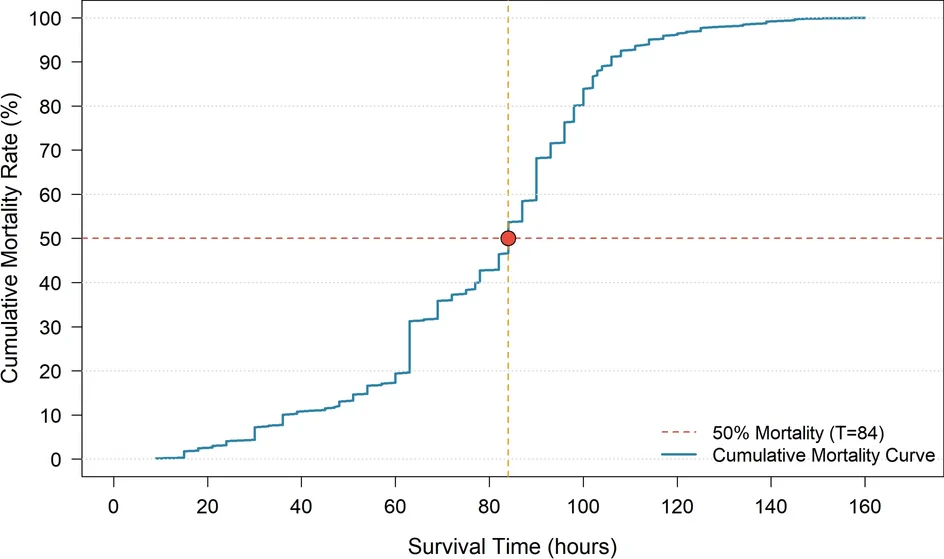
Cumulative mortality curve of the experimental population under acute ammonia nitrogen stress

Resistance to high ammonia nitrogen was measured as survival time. Initial mortality was observed at 9 h post-exposure to acute ammonia nitrogen stress, and all shrimp had died by 160 h. The median lethal time was determined to be 84 h for the entire population and 93 h for the sequencing population. The specific death circumstances are in Fig. 2. We calculated the mean survival time for each family within the sequenced population ([Additional file 1, Figure S1]). The results showed significant inter-family differentiation in the mean survival time overall. The mean values ranged from 63.67 to 105.33 h, with an overall mean of 89.16 ± 12.96 h. The maximum difference in mean survival time between families reached 41.66 h, indicating that different families possess distinct genetic or environmental differentiation potential for the survival time trait.

### Genotyping results and population structure

**Fig. 3:**
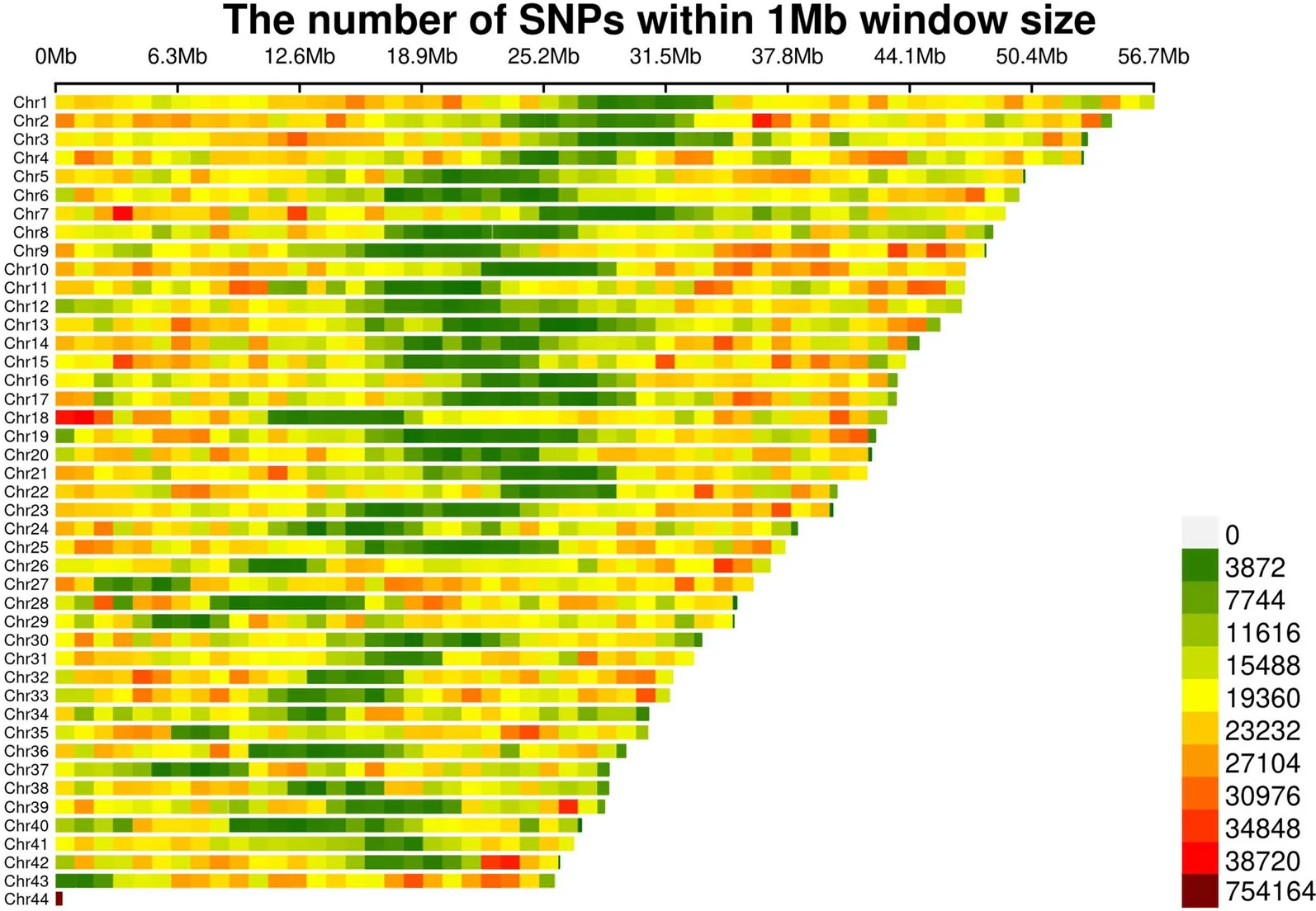
Genome-wide distribution of the 15,928,425 high-quality SNPs retained after quality control

**Fig. 4:**
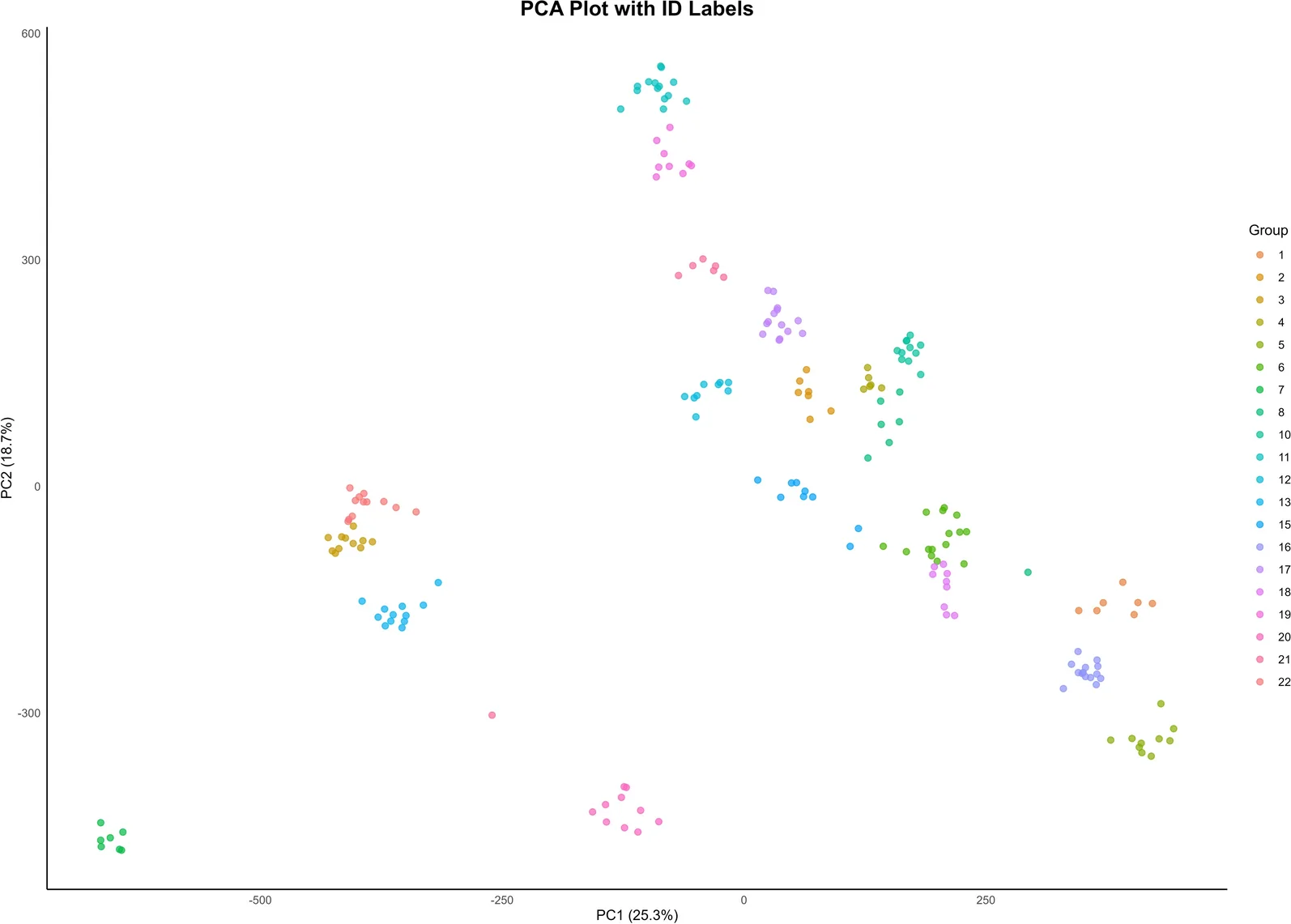
Principal component analysis (PCA) plot of the first two PCs showing genetic relatedness among individuals; PC1 and PC2 represent the first and second principal components, respectively; each color represents a different family

After whole-genome resequencing and quality control, we ultimately retained 15,928,425 high-quality SNPs, with a mean genotype call rate > 0.9, a minor allele frequency (MAF) *≥ * 1%, and no significant deviation from Hardy-Weinberg equilibrium (HWE $\textit{p} < 1 \times 10^{-5}$). These SNPs were evenly distributed across all chromosomal scaffolds (Fig. 3). The PCA plot revealed a clear population structure among the 200 shrimp from 20 full-sib families (Fig. 4), with each family clustering independently and thus representing distinct genetic subgroups in the study population.

### Genetic parameter estimation

The heritability estimate of the target trait derived from the pedigree-based additive relationship matrix ($\mathbf{A}$ matrix) in the PBLUP model was 0.26 ± 0.09 (Table 1). In contrast, all models integrating genomic information produced markedly higher heritability estimates, with values ranging from 0.56 ± 0.17 to 0.58 ± 0.17. Specifically, the GBLUP model based on the genomic relationship matrix ($\mathbf{G}$ matrix) yielded a heritability of 0.57 ± 0.16, the ssGBLUP model using the combined pedigree-genomic relationship matrix ($\mathbf{H}$ matrix) generated a heritability of 0.57 ± 0.17, and the four Bayesian variable selection models (Bayes A, Bayes B, Bayes C*π *, Bayesian LASSO) returned heritability estimates of 0.58 ± 0.16, 0.58 ± 0.15, 0.56 ± 0.17 and 0.58 ± 0.17, respectively (Table 1).

**Table 1:** Heritability, predictive accuracy and bias of different models

Model	$h^2 \pm$ SE	Prediction accuracy (SE)	SD	Min	Median	Max	Bias (SE)
PBLUP	0.257 ± 0.094	0.333 ± 0.041	0.183	0.151	0.299	0.623	1.237 ± 0.285
Bayes A	0.577 ± 0.164	0.462 ± 0.035	0.156	0.251	0.409	0.753	1.096 ± 0.211
Bayes B	0.579 ± 0.153	0.461 ± 0.036	0.160	0.302	0.391	0.799	1.097 ± 0.211
Bayes C*π *	0.564 ± 0.171	0.455 ± 0.034	0.152	0.312	0.386	0.773	1.080 ± 0.221
Bayesian LASSO	0.582 ± 0.170	0.464 ± 0.028	0.123	0.315	0.447	0.801	1.191 ± 0.200
GBLUP	0.568 ± 0.163	0.441 ± 0.059	0.263	0.133	0.426	0.788	1.133 ± 0.259
ssGBLUP	0.566 ± 0.165	0.458 ± 0.057	0.253	0.139	0.462	0.790	1.140 ± 0.151

SD: Variability across cross-validation (CV) replicates; Min: Minimum prediction accuracy; Median: Median prediction accuracy; Max: Maximum prediction accuracy

### Predictive ability of different prediction models

**Fig. 5:**
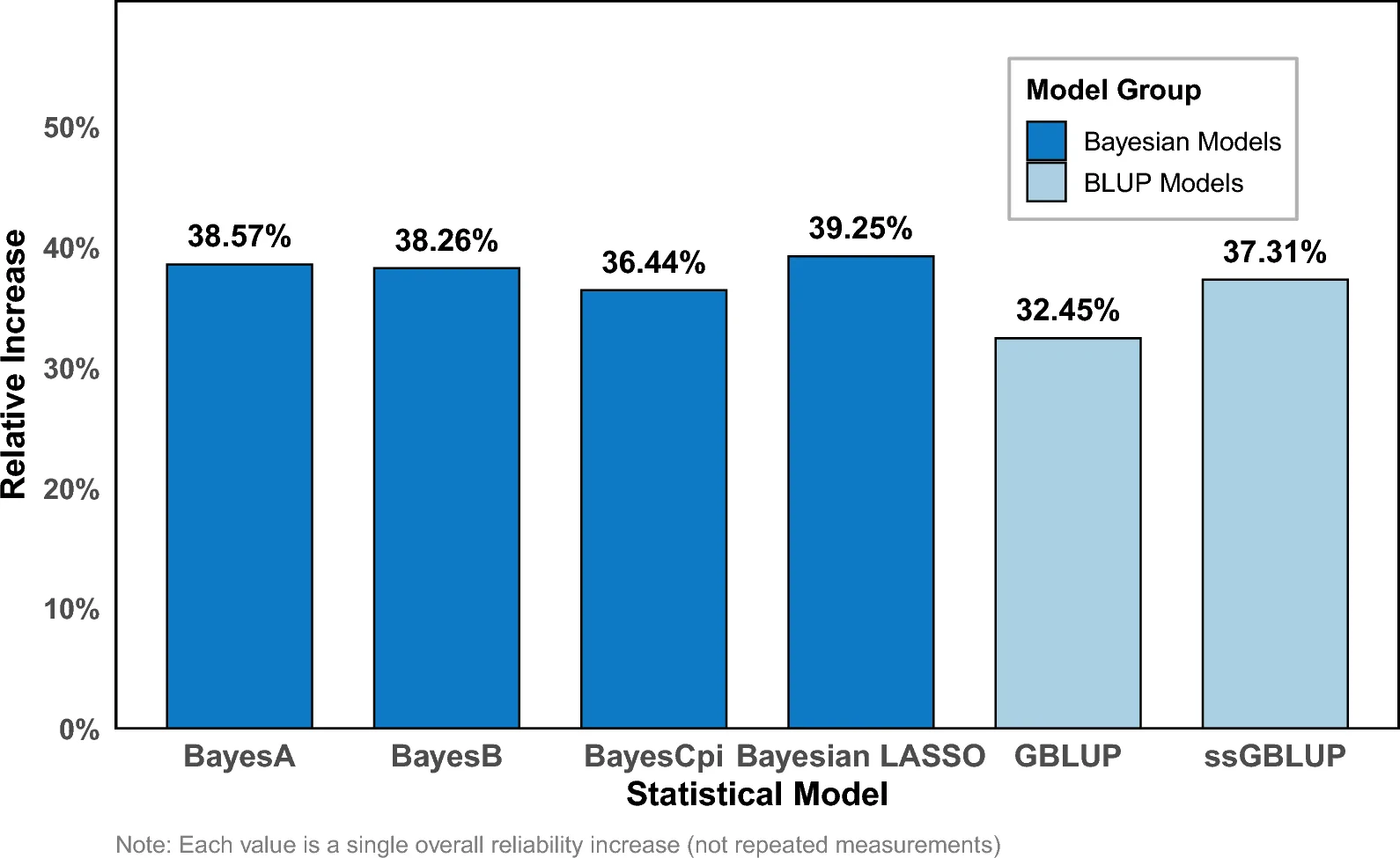
Relative improvement (%) in prediction accuracy of six genomic selection (GS) models compared with pedigree-based best linear unbiased prediction (PBLUP). Positive values indicate higher prediction accuracy than PBLUP

Using five-fold cross-validation, we compared the predictive accuracy of the seven models (Table 1). All six GS models were more accurate than the PBLUP model. GS models showed 32.45% to 39.25% improvement in accuracy over PBLUP (Fig. 5). Among them, the ssGBLUP model in the direct method and the Bayesian LASSO model in the indirect method showed the highest improvement in accuracy.

### Correlation between different prediction models

**Fig. 6:**
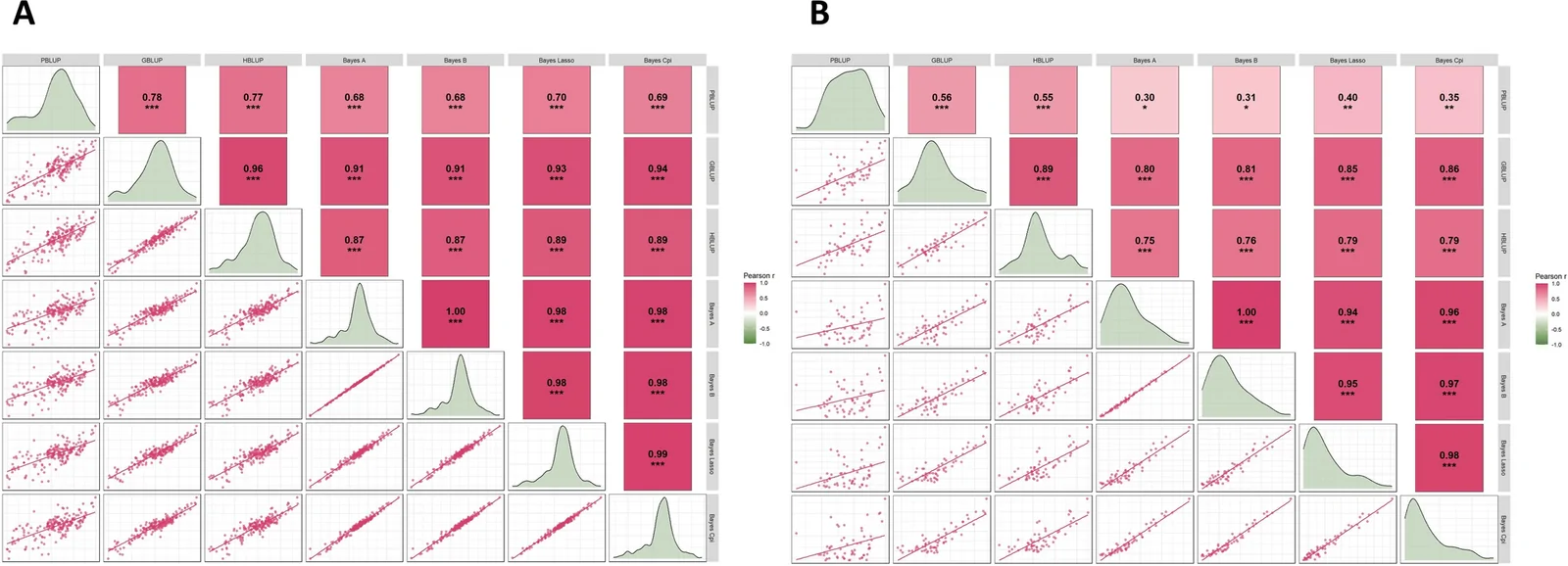
Pearson correlation of estimated breeding values from different models. **A** All individuals (n=200); **B** Top 30% of individuals with longest survival time (n=60). The panels below the diagonal are scatter plots of the linear fit for breeding values between models. The panels above the diagonal display the corresponding correlation coefficients (*** extremely significant; ** highly significant correlation; *significant correlation). The diagonal itself presents the kernel density plots for the distribution of breeding values for each model

Inter-model correlation matrices across prediction models are presented in Fig. 6A. The results indicate that the GEBVs from all the GS models were highly correlated, with coefficients from 0.87 to 1.00. In particular, the predictions from the four Bayesian models were nearly identical (r>0.98). In contrast, the correlation coefficient between PBLUP and all GS models was relatively lower, ranging from 0.68 to 0.78. When considering only the top 30% of individuals, the correlations between EBVs from PBLUP and GEBVs from the GS models decreased substantially compared to the full-population analysis, with Pearson correlations ranging from 0.30 to 0.56. In contrast, the correlations among the six GS models remained high but were slightly lower than those observed for the entire population, with Pearson correlations between 0.75 and 1.00 (Fig. 6B).

### Validation of GS in progeny

**Fig. 7:**
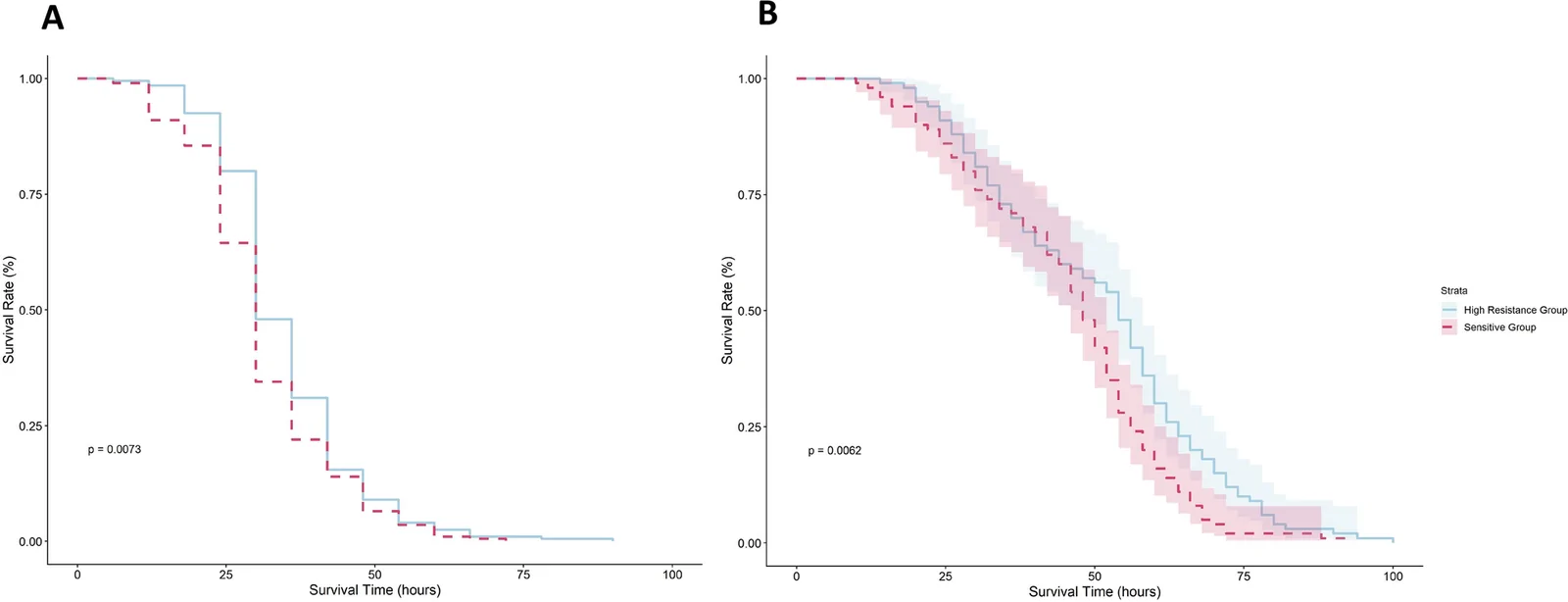
Kaplan-Meier survival curves for the high-resistance and sensitive groups in the two-stage progeny validation experiment; **A** First-stage ammonia nitrogen challenge. **B** Second-stage ammonia nitrogen challenge

A two-stage experimental design was implemented to evaluate ammonia nitrogen stress resistance in *L. vannamei* populations stratified into high resistance and sensitive groups. Comparative performance analysis revealed that the performance of shrimp in the high resistance groups was significantly better than that of the sensitive groups in both experimental stages, with improvements of 11.82% and 11.38%, respectively. The results of Kaplan-Meier survival curves (Fig. 7) confirmed a highly significant difference in survival time between the two groups in the first stage ($\chi ^2 = 7.1901, \textit{p} = 0.0073$). The high-resistance group exhibited a markedly longer mean survival time (35.01 h) compared to the sensitive group (31.32 h), with a hazard ratio (HR) of 1.118, indicating an 11.8% survival advantage. This finding was also validated in the second stage, where the difference remained highly significant ($\chi ^2 = 7.4943, \textit{p} = 0.0062$). The high-resistance group showed a longer mean survival time (50.94 h) compared with 45.72 h in the sensitive group with an HR of 1.114. The correlation between GEBVs and the actual survival time phenotypes of the validation population was statistically significant, exhibiting a weak-to-moderate positive association (r = 0.306, *p* < 0.05).

## Discussion

### Heritability of high ammonia nitrogen resistance trait

The heritability estimated using PBLUP in this study was 0.26 ± 0.09, which is relatively close to the 0.17 ± 0.08 previously estimated by Yuan et al. [12] The slight difference between the two studies may be related to differences in population background, family structure, and experimental conditions. The use of genomic data in this study captured a significantly higher heritability ($h^2$=0.56−0.58). This is consistent with the findings on the resistance of *L. vannamei* to WSSV [30]. Similar patterns, where GS models yield higher heritability estimates than pedigree-based models, have also been observed in *Fenneropenaeus chinensis* [40], *Macrobrachium rosenbergii* [41], *S. salar* [22], *O. mykiss* [23], and *Clarias macrocephalus* [42].

The inclusion of genomic information improves the estimation of genetic variance components and heritability. This is primarily because pedigree-based relationship matrices describe the expected genetic relationships among individuals based on recorded ancestry, whereas genomic relationship matrices constructed from genome-wide SNP markers estimate realized genetic relationships based on marker similarity among individuals [19, 35]. Consequently, GS models can account for Mendelian sampling variation among full-sib and half-sib relatives that cannot be captured by pedigree-based relationship matrices [36]. By providing a more precise representation of the true genetic relatedness among individuals, genomic information facilitates a better separation of additive genetic variance from environmental and residual variance, thereby reducing potential confounding between these variance components and improving the estimation of heritability.

In addition, GS models can better capture the cumulative effects of numerous small-effect loci distributed across the genome, which is particularly relevant for complex polygenic traits[22, 43]. In the present study, the GS models yielded heritability estimates that were higher than those obtained from PBLUP. This indicates that a substantial proportion of the additive genetic variation underlying ammonia nitrogen resistance was more effectively captured when genomic information was incorporated. These findings support the use of genomic information to improve the efficiency of breeding programs targeting ammonia nitrogen resistance in *L. vannamei*.

In our study, survival time (a continuous trait) was employed as the phenotypic variable for genetic and genomic analyses, rather than survival status (a binary trait). This choice was mainly motivated by the challenges associated with binary trait analysis in relatively small datasets, as described in the threshold model framework [44]. Binary traits convert continuous phenotypes into discrete categories, which may reduce the amount of phenotypic information available for analysis. Under relatively small sample sizes, this can affect the precision of genetic parameter estimation and consequently influence the performance of genetic evaluation and GS. For this reason, we adopted survival time, a continuous phenotype that preserves full phenotypic variation and supports more accurate estimation of genetic parameters. The issue of limited sample size can be addressed in future large-scale breeding programs. The widespread use of breeding chips has greatly reduced genotyping costs, making it feasible to genotype large populations. In this context, integrating both continuous survival time and binary survival status into GS models may further improve selection efficiency.

### Comparison of predictive abilities of different models

In this study, cross-validation regression analysis of predicted phenotypes showed that the predictive abilities of the six GS models were not significantly different, all significantly higher than those based on pedigree models, this is attributed to the use of genetic variations associated with SNPs. The 32.45% to 39.25% gain in accuracy is significant and agrees with similar studies in aquatic species[32, 41, 45–47]. This supports the growing evidence that GS is a valuable tool for improving complex traits in aquaculture.

It should be mentioned that there were only small differences in performance between the six GS models. Bayesian models performed slightly better than BLUP models, but not by much. This usually happens when a trait is controlled by a large number of genes with small effects. Previous GWAS studies on *L. vannamei* suggest that there are no major genes controlling ammonia nitrogen resistance[17]. For such traits, the simpler assumptions of GBLUP are almost as effective as the more complex ones in Bayesian models. For practical breeding programs, this means that computationally faster models such as GBLUP and ssGBLUP are excellent choices. This is also why the ssGBLUP results were chosen for the validation of progeny in this study. The Bayesian method, with its prior that most SNPs are ineffective while a few have large effects, is particularly suitable for traits influenced by large-effect quantitative trait locus(QTL). This is evident in disease resistance traits in Atlantic salmon and rainbow trout, where Bayesian models have demonstrated significantly higher predictive accuracy compared to BLUP models [48, 49].

Moreover, high correlation coefficients (ranging from 0.87 to 1.00) were observed among all GS models, further indicating that these GS models exhibit consistent predictive performance. Similar findings were observed in studies on *Vibriosis* resistance trait in *Crassostrea gigas* [45] and *Piscirickettsia salmonis* [20] resistance trait in *S. salar*. Bangera et al. [20] also reported relatively lower correlations (0.76 to 0.81) between PBLUP and GS models in *S. salar*, which is consistent with the results of this study (0.68 to 0.78). This suggests that GS models and PBLUP models may differ in their predictions of progeny breeding values. The decline in correlation between PBLUP and genomic models for the top 30% of individuals highlights a key practical implication: when selecting breeding candidates under a realistic selection intensity, the ranking decisions derived from pedigree information alone may differ substantially from those based on genomic information. This divergence can lead to different selection outcomes and potentially slower genetic progress if only traditional methods are used. On the other hand, the consistently high correlations among all genomic models for the top-ranked individuals suggest that these models converge on a similar set of elite candidates, reinforcing the robustness of genomic predictions.

### Validation of GS results

In the past, many studies evaluated the performance of GS using only cross-validation. In the present study, prediction accuracies were initially assessed using random five-fold cross-validation repeated for 20 replicates, this repeated random partitioning reduces sampling error and improves the robustness of the results. Moreover, cross-validation maximizes the use of available data and provides a convenient framework for comparing prediction models. Despite these advantages, cross-validation has several limitations. One important concern is that closely related individuals from the same full-sib or half-sib families may be represented in both the training and validation sets. Consequently, genomic relationships shared between the two subsets can inflate the observed prediction accuracy, resulting in more optimistic estimates than would be expected in truly independent populations [48, 50, 51]. In addition, the predictive ability estimated by cross-validation is influenced by the heritability of the target trait, which may further complicate comparisons among studies and traits [48].Previous studies have demonstrated that prediction performance is strongly dependent on the genetic relationship between training and validation populations, with higher accuracies generally observed when closely related individuals are included in both groups [49, 52, 53]. Therefore, the prediction accuracies obtained from cross-validation should be interpreted primarily as estimates of within-population predictive performance rather than as direct indicators of prediction accuracy in future breeding generations. For commercial breeding programs, independent validation is essential for determining whether the predictive ability observed in cross-validation can be maintained under practical breeding conditions. Progeny testing remains one of the most reliable approaches for validating genomic predictions, and recent studies have increasingly incorporated comparisons of progeny performance to confirm the effectiveness of GS [30, 40, 45, 54, 55]. This independent evaluation effectively compensates for the inherent limitations of cross-validation by assessing the performance of selected families under actual farming conditions, thereby providing a more realistic assessment of GS performance.

Among *L. vannamei*, Lillehammer et al. [30] and Liu et al. [31]conducted progeny validation of WSSV resistance traits and AHPND resistance traits, They both found that under challenge conditions, the survival rate of the resistant groups was significantly higher than that of the sensitive groups. In this study, we also performed a progeny validation for the trait of ammonia nitrogen resistance in* L. vannamei*. Unlike previous studies, this study combined predictions from a GS model with the BLUP selection method. We designed two different mating combinations at the family level: high resistance groups and sensitive groups. The efficacy of GS was then validated in the next generation using a two-stage stress trial.

In our study, the consistent results of the Log-rank tests across both experimental stages conclusively demonstrate that the high-resistance group has a significantly longer survival time than the sensitive group, confirming the reliability and stability of our findings. Although this improvement is not as large as those reported for conventional GS in other studies (e.g., 18.42% for Vibriosis resistance in Pacific oysters[45] and 27.8% for visceral white-nodules disease resistance in large yellow croaker [54]), it still represents a significant difference, confirming the reliability of this study. This conclusion is further supported by the significant positive correlation (r = 0.306) observed between the GEBVs and the actual phenotypes in the progeny population.

GS was first and most widely applied in livestock [56], where animals have high individual value and are easy to mark, allowing the technology to develop rapidly. In contrast, shrimp are small in size, making it hard to mark them in the early stages. Moreover, a single female shrimp can produce up to 200,000 eggs at a time. While this ensures the high value of broodstock, it also makes it hard to establish a selection population, as deciding which individuals to select for genotyping is difficult, and genotyping all progeny is expensive.

Based on these practical concerns, we designed and tested a breeding strategy that combines the BLUP method with GS. Progeny trials confirmed that this approach is viable for commercial use. While its accuracy is slightly lower than conventional GS approaches, it offers major practical advantages: it eliminates the costly need to sequence the entire candidate population, and it will reduce the negative impact on other important traits that often comes with intense selective breeding.

## Conclusions

This study applied GS models to improve ammonia nitrogen resistance in *L. vannamei* and achieved a 32.45% to 39.25% enhancement in predictive accuracy compared to traditional PBLUP. We proposed a transitional approach combining BLUP method with GS to promote the application of GS in commercial breeding. Progeny testing under high ammonia nitrogen stress showed that the high resistance groups established using this method exhibited significantly higher survival time than the sensitive groups. Given the current constraints of high sequencing costs and the lack of a comprehensive multi-trait breeding system in *L. vannamei*, the breeding method combining BLUP method with GS offers a practical and temporarily viable alternative to conventional GS in commercial breeding programs.

## Electronic Supplementary Material

Below is the link to the electronic supplementary material.

Figure S1. Mean survival time of each family within the sequenced population.

## Data Availability

The data presented in the study have been deposited in the NCBI repository, accession number: PRJNA1308829
